# Characterization and phylogenetic analysis of the complete chloroplast genome of *Cercis canadensis* ‘Forest Pansy’

**DOI:** 10.1080/23802359.2019.1698359

**Published:** 2019-12-13

**Authors:** Lijuan Feng, Jihan Tao, Xuemei Yang, Qiqing Jiao, Chuanzeng Wang, Yun Cheng, Yanlei Yin

**Affiliations:** a Shandong Institute of Pomology, Taian, Shandong, China;; b Shandong Academy of Agricultural Sciences, Jinan, Shandong, China;; c Liaocheng Natural Resources and Planning Bureau, Liaocheng, Shandong, China

**Keywords:** *Cercis canadensis*, Forest Pansy, chloroplast genome, phylogenetic relationship

## Abstract

*Cercis canadensis* ‘Forest Pansy’ is a tree species with high ornamental value, which complete chloroplast (cp) genome was sequenced, assembled, and annotated. The genome size is 158,960 bp with a total GC content of 36.17%. The cp genome is made up of a large single-copy region (88,114 bp), a small single-copy region (19,590 bp), and two inverted repeat regions (25,628 bp each). It contains 128 genes, including 84 protein-coding genes, 36 tRNA genes, and 8 rRNA genes. Eighteen genes were duplicated in IRs. The maximum-likelihood (ML) phylogenetic analysis indicated that the Leguminosae species are grouped together, and C. canadensis ‘Forest Pansy’ is closely related to *C. canadensis*. The result would provide valuable information for genetic studies on *Cercis genus*.

‘Forest Pansy’ is a variant of the *Cercis canadensis*, which belong to *Cercis* of Leguminosae family (Davis et al. 2002). It is deciduous shrubs or small landscape tree, which exhibits considerable morphological diversity, including variation in plant architecture, plant size, and flower and leaf colors (Roberts et al. 2015). It is native to Canada, and is mainly distributed in the USA, China, and Africa (Zhang et al. 2009). It has higher ornamental value with beautiful pink flowers and purple leaves. It is widely cultivated in botanical gardens, parks, and on the roadside in China (Li et al. 2017). In this present study, we reported and characterized the complete chloroplast (cp) genome of Forest Pansy based on Illumina pair-end sequencing and compared it with other genus cp genome sequences. The result would supply valuable information for the evolution process and conservation genetics of *C. canadensis*.

The fresh leaves of *C. canadensis* ‘Forest Pansy’ (Voucher specimen Accession No. SDJNDZJ0069) was collected from the Taidong field of Shandong Institute of Pomology (36.20°N, 117.12°E), Shandong Province, China. Total genomic DNA was extracted using the DNeasy Plant Mini Kit (Qiagen, Venlo, Netherlands). cpDNA sequencing was performed with an Illumina Hiseq 2500 platform by Nanjing Genepioneer Biotechnologies (Nanjing, China). The raw paired-end reads of cpDNA were filtered using fastp program (Chen et al. 2018), and *de novo* assembly performed using GetOrganelle (Jin et al. 2018). The cp genome was annotated using the program DOGMA (Wyman et al. 2004), with the cp genome of *Cercis glabra* (GenBank Accession No. KY806281) (Wang et al. 2017) serving as the reference. A circular genome map of the genome was generated with OGDRAW (http://ogdraw.mpimp -golm.mpg.de/) (Lohse et al. 2013). The complete cp genome was deposited in the GenBank (Accession: MN562096).

The complete cp genome of *C. canadensis* ‘Forest Pansy’ was 158,960 bp in length with a total GC content of 36.17%. It is shorter than the reference species *Cercis glabra* (159,181 bp) and exhibited a typical quadripartite structure with known *Cercis* cp genomes. The cp genome is made up of a large single-copy region (LSC) of 88,114 bp, a small single-copy region (SSC) of 19,590 bp, and two inverted repeat regions (IRs) of 25,628 bp each. The complete cp genome encoded 128 unique genes, which contained 84 protein-coding genes, 36 transfer RNA (tRNA) genes, and 8 ribosomal RNA (rRNA) genes. The tRNA genes are distributed throughout the genome with 21 in the LSC, one in the SSC, and 14 in the IR regions, while rRNAs are only situated in the IR regions. There were 18 duplicated genes in IRs, including seven protein-coding genes (*rpl2*, *rpl23*, *ndhB*, *rps7*, *rps12*, *ycf2*, and *ycf15*), seven tRNA genes (*trnICAU*, *trnL-CAA*, *trnV-GAC*, *trnI-GAU*, *trnA-UGC*, *trnRACG*, and *trnN-GUU*) and four rRNA genes (*rrn16*, *rrn23*, *rrn4.5*, and *rrn5*). Among the protein-coding genes, two genes (*clpP*, *ycf3*) contained two introns, and other eight genes (*atpF*, *ndhA*, *ndhB*, *petB*, *petD*, *rpl2*, *rpoC1*, and *rps12*) had one intron each.

To ascertain the phylogenetic position of *C. canadensis* ‘Forest Pansy’, 25 complete cp genomes within Leguminosae family were selected, *Fraxinus excelsior* (GenBank Accession No. NC_037446.1) and *Platanus occidentalis* (GenBank Accession No. DQ923116.1) as outgroup. The cp genomes of these species were aligned Using MAFFT v7.3 (Kazutaka and Standley 2013). The maximum-likelihood (ML) phylogenetic tree was constructed by the IQ-TREE with the best-fit model identified using ModelFinder (Kalyaanamoorthy et al. 2017). The result showed that the Leguminosae species are grouped together, and *C. canadensis* ‘Forest Pansy’ is closely related to *C. canadensis* (Figure 1). It will be valuable for the genetic study on *Cercis* genus.

**Figure 1.**
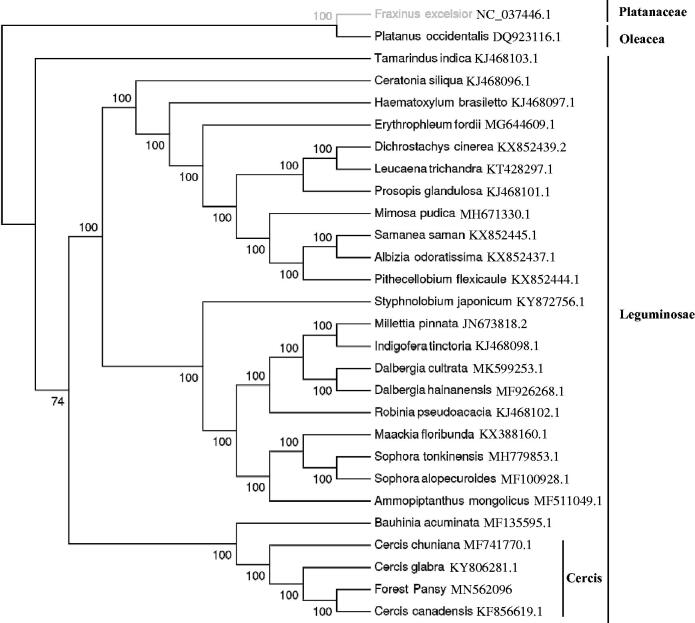
The best maximum-likelihood (ML) phylogenetic tree based on the 28 complete chloroplast genome sequences. The number on each node indicates bootstrap support value.
